# How Did the Pandemic Affect Communication in Clinical Settings? A Qualitative Study with Critical and Emergency Care Nurses

**DOI:** 10.3390/healthcare10020373

**Published:** 2022-02-14

**Authors:** José Luis Díaz-Agea, Irene Orcajada-Muñoz, César Leal-Costa, Maria Gracia Adánez-Martínez, Adriana Catarina De Souza Oliveira, Andrés Rojo-Rojo

**Affiliations:** 1 Faculty of Nursing, Universidad Católica de Murcia, 30107 Guadalupe, Spain; jluis@ucam.edu (J.L.D.-A.); iorcajada91@gmail.com (I.O.-M.); acatarina@ucam.edu (A.C.D.S.O.); arojo@ucam.edu (A.R.-R.); 2 Faculty of Nursing, Universidad de Murcia, 30120 El Palmar, Spain; 3 Faculty of Medicine, Universidad de Murcia, 30120 El Palmar, Spain

**Keywords:** health communication, COVID-19, critical and emergency care, qualitative research

## Abstract

The objective of the present study was to determine the subjective impact of the pandemic due to COVID-19 on communication, as perceived by nurses working at emergency services and Intensive Care Units at various hospitals in the Region of Murcia (Spain). A qualitative study was conducted based on the content analysis of 12 semi-structured individual interviews. The participant recruitment process was performed through a snowball sampling technique. Four main dimensions, eleven categories, and two sub-categories were obtained: (1) communication (communicative expressions, both verbal and non-verbal-, and limitations); (2) emotional aspects (positive, negative); (3) overload (first wave, second wave, and third wave); and (4) relationships (health professionals–patients, healthcare professionals, patients–family, and family–health professionals). The main findings of the study show that communication was slightly affected during the pandemic, especially the non-verbal kind, with verbal communication maintained and, in some occasions, strengthened. The lack of training in communication skills and its influence on the management of difficult periods was another important finding. Communication in general deteriorated during the pandemic, especially during the initial waves. Non-verbal communication was more affected due to the use of Personal Protective Equipment and the initial fear of infection, with this finding strongly observed in departments such as emergencies or critical care. The nurses who were interviewed underlined negative emotional aspects associated with a deficit in communication. The positive aspects described were associated with the creation of mutual support spaces and the group cohesion of the work teams during the pandemic. As an implication for current and future clinical practice, we recommend a coordinated institutional response to mitigate the potential emotional effects on workers by designing appropriate communication and emotional expression protocols.

## 1. Introduction

In March 2020, the World Health Organization (WHO) declared the COVID-19 as a pandemic. This disease has had a great impact on morbidity and mortality worldwide, and the level of infection overloaded health systems, resulting in diverse alterations in care provision [1]. Since the first case in Spain was detected, the increase in the number of people diagnosed and deceased has not stopped rising [2]. The health crisis caused by COVID-19 has had important consequences in Spain [3,4,5], with this country being among the countries most affected by COVID-19 worldwide, especially during the first and sixth waves [6]. It is evident that the data are discouraging but even more so if we observe the diagnosed cases of healthcare personnel [7]. Studies on the impact of the pandemic on health workers in the Spanish context have shown a high prevalence of emotional and general health symptoms [8,9]. In the region of Murcia, to date, 331,577 confirmed cases of COVID-19 have been registered. Epidemiological discharge has been given to 302,303 patients, and 2010 deaths have occurred [10].

Communication has played a fundamental role in health centers and hospitals in the management of this health problem and the propagation of the virus although it has been difficult due to the work setting at these centers and the use of masks [11,12]. Communication plays a primordial role on the interactions of people in clinical settings, and we must therefore consider the importance of correct and efficient communication. This communication must be suitable to the needs of the patients, their families, and health professionals [13]. Communication skills are a key competency for nurses, as it is indispensable for establishing a relationship of support, safety, and quality of care provided to patients [14]. Good communication has a positive influence on the perception of the health system users and their families, and it favors the management of the patient’s health, adherence to a therapy, the solution of problems, and emotional support (such as how to face situations of mourning) [11,13,15]. Studies conducted on health professionals and communication skills can be equated to investing in better patient care [16]. It has also been observed that good communication skills are fundamental for preventing the burnout syndrome found in health professionals [17,18,19,20]. Furthermore, communication failures are the leading cause of inadvertent patient harm [21].

With special emphasis on the current pandemic, communication can have different effects on the emitter and the receiver, depending on the way it is conducted. For example, for the receiver, adequate communication can help in the process of acceptance and adaptation to the new reality in situations of mourning although communication skills and the management of basic operating guidelines are needed, which point to the need for training. For the emitter, effective communication can imply a decrease in the levels of stress, which can reduce the probability of suffering from burnout [17]. Therefore, communication is a process of reciprocal interaction, which benefits both nurses and patients [22,23].

Communication with critical COVID-19 patients is more complicated given that many of them are sedated, intubated, or their lives are at risk. We can also include the loneliness they experience, as they cannot be accompanied by their family. These patients experience feelings and emotions, such as pain, fear, anguish, sadness, etc., and appropriate communication skills of health professionals are therefore needed [24,25]. According to the WHO, good communication has become as essential as epidemiological training and laboratory analyses for the control of COVID-19 outbreaks [26].

There are two types of communication, namely verbal and non-verbal. A greater importance is usually granted to verbal communication, but studies have shown that more than 55% of communication is conducted through non-verbal means [27,28]. Non-verbal communication between individuals does not involve the content of spoken language. Instead, it is based on the unspoken language of facial expressions, eye contact, and body language [29]. For this, we must consider that the continued use of surgical masks could significantly increase the difficulties in non-verbal communication, especially because facial gestures practically disappear [30]. Furthermore, we must consider people who have some type of disability or limitation for communicating, such as deafness, for whom non-verbal communication is indispensable, thereby granting more importance to facial gestures and lip-reading. In these cases, it is vital to consider resources that decrease these difficulties, such as communication boards and gestures of support [11].

The main objective of the study was to determine the subjective impact of the COVID-19 pandemic on communication, as perceived by nurses at hospital emergency services and Intensive Care Units (ICU) at various hospitals in the region of Murcia (Spain).

The specific objectives were to analyze verbal and non-verbal communication perceived by health care professionals and to identify the possible limitations they found; to identify emotional aspects, both positive and negative, observed by health professionals during the pandemic, associated with communication; to explore the overload experienced by health professionals in the different stages of the pandemic as well as the associated feelings; and to describe, from the point of view of the nurses, the relationships between health professionals, patients, and families since the start of the pandemic.

## 2. Materials and Methods

### 2.1. Design

A qualitative study was designed based on the analysis of individual, semi-structured interviews, which were conducted to delve into the characteristics of communication perceived by nurses working at emergency services and ICUs at different hospitals in the region of Murcia (Spain). We sought to individually identify the main dimensions referred to in the study objectives through the interviews given to the participants.

### 2.2. Study Setting and Participants

To recruit informants who would be ideal for the study, an intentional, non-probabilistic sampling method was utilized. The participants were health professionals who met the following inclusion criteria: (1) nurses who had worked during the pandemic in services in which they had been in contact with COVID-positive patients, especially in emergency services and ICUs and (2) the participant’s consent for the study.

The participant recruitment process was conducted through snowball sampling, resulting in 12 volunteers (Table 1). The internal composition met the homogeneity criteria (all of the participants met the inclusion criteria) and the heterogeneity criteria (as the representation of different opinions were sought, the nurses had to come from different services and hospitals in the region of Murcia, Spain). The number of participants was chosen according to the saturation criterion [31]. Information saturation refers to the moment in which qualitative material ceases to provide new data after conducting several interviews. At this moment, researchers stop collecting information. In qualitative research, the number of participants is not as relevant as in quantitative studies; nor is statistical representativeness expected. What is sought in qualitative research is depth, and what is fundamental is the quality of the participants’ contributions and the extent to which they help us to understand the phenomenon under study. Other qualitative studies similar to ours (referring to the subjective perspectives of health professionals on the pandemic) have been carried out with a similar number of participants [32,33]. When the phenomenon under study is striking, and the shared experiences are common, saturation is often reached with a small number of interviews, as evidenced in other qualitative studies [34,35].

### 2.3. Data Collection

The data were collected between the months of April and July 2021. Twelve semi-structured in-depth interviews were conducted. Each interview lasted an average of 38.52 min and was conducted by a member of the research team (IOM). The interviewer had experience in qualitative research and was conducting research in aspects of clinical communication for her doctoral thesis. As this study was conducted in Spain, the language used in the interviews was Spanish (Castilian). A professional native translator (MF) oversaw the translation of the excerpts from the interviews for this manuscript. The role of the interviewer was to guide the interview towards the main objectives of the study as well as to re-guide the interview when the conversation deviated from the main aspects of the study.

At the start of the interview, the main researcher ensured that all the participants understood the aim of the study and what was required of them. The concepts of “communication” and “communication skills” were also clarified for them so that they would have a clear idea about the topics to be discussed during the interview. The following personal data were collected: age, professional category, hospital service in which they were currently employed, services in which they had previously worked, year of graduation from university, and years of experience. Next, the open-ended questions were presented, which had been agreed upon by the research team and contained the following topics (Table 2).

The interviews were face-to-face and were recorded on a digital audio/video recording device. The setting in which the interview took place was relaxing and comfortable, with all the safety measures in place (both the interviewer and each participant wore an FFP2 mask in addition to keeping an interpersonal distance of 2 m). The conversations were recorded and saved in a video/audio file for their later transcription and analysis. About 462.32 min of conversations were recorded.

### 2.4. Data Analysis

The files were transcribed verbatim into a single document that served as the basis for independent close reading by two different members of the research team with previous qualitative research experience (C.L.C. has a Ph.D. in Psychology and J.L.D.A. a Ph.D. in Social Anthropology). The two members of the research team independently read the document several times to obtain an overview of the content and identify key concepts. They then met to discuss the results and agree on a coding system to identify themes and sub-themes in the material. A careful examination of the data was undertaken to identify and conceptualize the meanings contained in the text. The units of meaning that emerged in the independent analysis of the text were identified and coded and then grouped into subcategories and categories based on their similarity, using an inductive method characteristic of qualitative research. To analyze the data, the MaxQDA^®^ 2018 software was utilized. At all times, the research team sought to have a neutral attitude to decrease the possible impact of their subjectivity in the process of information collection. The methodological approach followed during the study was phenomenological [36]. The guidelines proposed by Colaizzi [37] were utilized to analyze the data. Moreover, the typical criterion of theoretical saturation of data [38] was utilized when selecting the number of interviews necessary (when the responses were sufficiently repeated to obtain common patterns). When reporting the data and structuring the report of this qualitative research, we followed the guidelines recommended by the methodological literature, which provides rigor to the study. These guidelines have been consolidated into recommendations and an internationally recognized checklist called COREQ (Consolidated criteria for Reporting Qualitative research) [39], which served as a guide for the present study.

### 2.5. Ethical Considerations

The study was approved by the Ethics Board from the San Antonio Catholic University of Murcia (ref. num: CE102103). Before the collection of data, all the participants were previously informed about the objectives of the study and the posterior use of them for the research study considering its voluntary character. All the participants provided their informed consent, which included their permission to be recorded. The participant’s contributions were codified to guarantee anonymity and confidentiality, which included the destruction of the recorded sessions after the collection of necessary data. This research was carried out following the recommendations of the Declaration of Helsinki [40]. Participants were assigned a number code, between N1 and N12, to ensure anonymity. Individual hospitals were given a numerical code (H1, H2, etc.) rather than a name to help protect privacy.

## 3. Results

Of the 12 participants, 7 were women and 5 men, with a mean age of 36.9 years old. All of them were working at services in which they would be in contact with COVID-positive patients: four in Emergencies, six in the COVID ICU, one in the COVID hospital unit (hospital units adapted to care for COVID-19 patients who are not in critical condition but who require hospitalization), and one in Emergency Radiology Diagnosis. Their professional experience ranged from 3 to 20 years, and all of them were working since the start of the pandemic. Table 1 shows the main characteristics of the participants (age, sex, workplace, and experience). In the region of Murcia, there are a total of 10 public hospitals, out of which five are general university hospitals, and five are county hospitals. The participants in our study worked at three general hospitals and two county hospitals.

**Figure 1:**
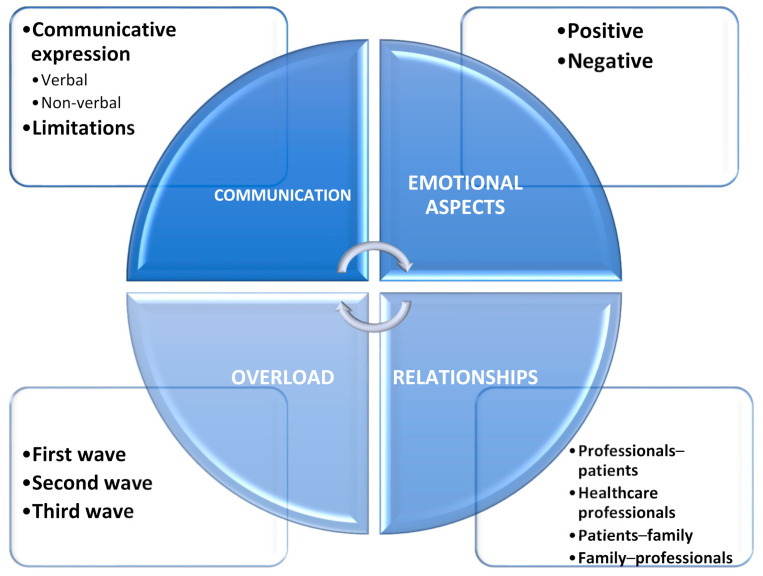
Dimensions, categories, and sub-categories.

Four main dimensions, eleven categories, and two sub-categories were obtained (Figure 1 and Table 3).

### 3.1. Dimension 1. Communication

Two categories were obtained from this dimension, namely communicative expression and limitations, which themselves were divided into two sub-categories, including verbal and non-verbal.

#### 3.1.1. Communicative Expression: Verbal

For the participants, verbal communication was affected, especially at the beginning of the pandemic, due to the use of personal protection equipment/PPE (mask, screen, robe, etc.) although it is precisely due to this that communication improved over time and was re-enforced between colleagues by the surge in empathetic feelings:

“(...) communication was easier between colleagues if you had previously met them before wearing the PPE. It is clear to me that verbal communication has suffered; especially at the beginning, it was more limited.”

(N1)

“I think it is difficult for the professional to give information and for the patient to receive it because of the PPE. However, I saw better communication between colleagues than, for example, with the family, which was the big problem at the beginning of the pandemic.”

(N12)

A great amount of personal communication was lost, as communication via the telephone to contact family members highly increased. The nurses believed that the patients received the same amount of information about their disease:

“Young people communicated better with their relatives than older people. Of course, I am referring to communication via telephone because communication in person has been lost.”

(N6)

The health professionals who worked directly with COVID-positive patients, at which time a colleague was present to “mirror” them, had a better work organization.

All the participants agreed in that communication in situations of emergency was not affected, independent of whether the patient was COVID-19 positive:

“(…) the work dynamic in emergency situations was good, because in the end, once the team does what needs to be done, it will always be backed by the team that is outside (…)”

(N7)

“(…) in situations of emergency, all of us wear our armor, and we have to take risks to save a life; in that situation, communication is adequate for the situation you are facing (…)”

(N3)

“(…) communication was increased to its highest degree (…)”

(N5)

#### 3.1.2. Communicative Expression: Non-Verbal

Non-verbal communication was more complicated due to the use of personal protection equipment (mask). Facial expressions were limited, so more attention was granted to the expression of the eyes or gestures.

The participants concluded that before the pandemic, they could better observe the mood of a colleague, as compared to the present time, due to the use of masks; nevertheless, at present, they could still detect the emotions of others as long as they focused their attention to the eyes and gestures:

“You don’t see the facial expression; with PPE, you can hardly perform non-verbal communication. I think you get more information with the eyes when you wear PPE.”

(N1)

“(…) I notice it in the eyes, by looking at my colleagues’ eyes, I can tell if they are OK or not…by the gestures, in the manner in which they sit or walk, in the way they say hi…in my case, I have learned how to look at my colleagues’ eyes, and tell if he is tired or angry…I have learned how to observe all the gestures and behaviors, the manner in which they talk, and especially the way they look, to know how they are feeling…it’s complicated, and I supposed that it depends on how observant you become (…)”

(N3)

“(...) now it’s all eyes and voice (...) communication is something basic, you have to work on it every day, whether it’s with your eyes, gestures or whatever (...)”

(N2)

#### 3.1.3. Limitations in Communication

Various limitations were found, among which we found elderly individuals who were deaf, people with language barriers, patients who were under non-invasive ventilation, patients in the process of weaning, patients who had undergone a tracheostomy, etc.:

“(…) what happened to me is that deaf-mute people came, and it was a real problem, because obviously a mute-deaf person will look at your lips, but since you are wearing a mask, they can’t.”

(N3)

All the interviewees agreed in that communication was more difficult with disabled patients (deaf patients cannot read lips). Other means of communication were utilized for better communication, such as gestures or using paper and pen to write down what they wanted to say:

“(…) it’s more difficult, and you also don’t have skills to tend to these patients, I’m not sure if what I transmitted got to them correctly.”

(N5)

“I noticed it was more difficult to communicate with people with disabilities, but we used slates or signs... you survive to understand the patient.”

(N7)

### 3.2. Dimension 2. Emotional Aspects

Two categories were obtained on this dimension, which were grouped into positive and negative aspects.

#### 3.2.1. Positive Aspects

In this section, the participants underlined positive feelings, such as camaraderie or the feeling of becoming closer to their colleagues, and other feelings were also found, such as the acquisition of new knowledge and the feeling of overcoming, the special feeling established with the “mirror” colleague, and feelings of solidarity and responsibility:

“(…) I felt completely committed to the cause, with solidarity. It has been very intense. The comradeship came to the surface, and I had the satisfaction of taking the work forward day by day (...) My conscience is clear. I have to say that I have learned a lot, and I have acquired new knowledge and incorporated it. I have also become aware of the responsibility we have.”

(N5)

“(…) I saw a high degree of camaraderie (…)”

(N10)

#### 3.2.2. Negative Aspects

The main negative feelings were fear, stress, and physical and psychological wear although other feelings predominated, such as uncertainty, insecurity, chaos, frustration, impotence, and worry:

“(…) it was like a war, but without knowing the enemy, you knew you had an enemy who could give you a deadly disease, then you would say: ‘well, what means do I have to face this’ (…) I was overcome with emotions, at first I cried a lot (…) The feeling was fear, always a lot of fear when thinking if I had become infected (…)”

(N5)

“(…) it’s hard for people to understand what we health workers went through; it would change a lot of behaviors in society in general.”

(N11)

### 3.3. Dimension 3. Overload

In this dimension, we obtained three categories, which were divided considering the three first waves of the pandemic in Spain.

#### 3.3.1. First Wave

The interviewees characterized the first wave with the lack of personal protection equipment (PPE) and information, in which they experienced a greater exposure, and it was defined as psychologically more difficult and chaotic, in which only the more urgent pathologies were tended to in person in Primary Care:

“(…) we were afraid of not knowing if we were doing things right (…)”

(N10)

“It was all speculation because nothing was known. There was a lack of information, we didn’t even know how to use a PPE. The health centers were closed, and we only attended emergencies, acute care, or wound cleaning. We were finding out new things every day. There was more information in primary care than in specialized care at the beginning. We did things like in other ICUs but without really knowing why... they didn’t train us...”

(N9)

#### 3.3.2. Second Wave

Those interviewed differentiated the second from the first wave, as they had more information, more equipment, and more experience. There was less fear and more protocols although these were still changing. The nurses became accustomed to wearing masks and PPE:

“Conditions improved over the following months. I learned to deal with stress, uncertainty, and chaos. New protocols were introduced (...) to this day.”

(N8)

“(…) dealing with the shifts was better with time and with the experience we gained, also gaining confidence little by little (…)”

(N2)

#### 3.3.3. Third Wave

The participants characterized the third wave as the hardest period of work due to the pressure on medical care although they were more accustomed to wearing PPE and worked faster. They had more knowledge, and the situation was more peaceful thanks to the start of vaccination. They agreed that they felt safer due to the use of the PPE:

“(…) although it was very, very hard, and perhaps the hardest from what was experienced in the entire region (…), but you face it differently.”

(N6)

“It was the hardest working period of all, but we had the situation under control.”

(N8)

“The key point was when we got vaccinated, it was a bit of a liberation.”

(N10)

### 3.4. Dimension 4. Relationships

In this dimension, we included four categories: health professionals–patients, between health professionals, family–patients, and family–health professional relationships.

#### 3.4.1. Health Professionals–Patient

All the participants agreed in that especially during the start of the pandemic, there was a loss of physical contact and much more limited communication due to the use of PPE, which were utilized specially to reduce the virus exposure time:

“You try to convey affection, but with PPE, it is more difficult. You spend less time in the rooms with the patient. You are left ‘affected’ after experiencing this, the suffering during the shift and the feeling of sorrow for not being able to save many patients.”

(N5)

With time, the relationships evolved given the increased knowledge and therefore increased safety. The general perception was that the patients received the same amount of information about their disease, without considering the emotional part (which was impaired). Communication with patients under non-invasive mechanical ventilation or with some type of disability was more difficult:

“(...) Fear and caution when approaching the patient (...) you thought of all of them as suspects of being infected or being infected yourself and infecting others. At the beginning, the relationship was more distant; now, a little less. In general, there was less communication with the patients, less face-to-face care, and more telephone care. With the patients who had BIPAP, it was more difficult to communicate.”

(N12)

“(…) at first, you did what you had to do, and you left the room, and now, thanks to the vaccination, we have lost some of the fear, you feel safer, and we have more information (…)”

(N2)

“(…) at first, you couldn’t make great efforts in patient care because you had to leave the room quickly, and now we know more (…)”

(N6)

“Communication has worsened because of the barriers. Patients were inside the box, and you had to talk to them from the other side of the door. We didn’t have the means to improve communication at the beginning (tablets or telephones inside the box), but when we went in to do something to the patient, we would explain or let them know that we were there to help them. Depending on what the patient asked you for, you gave them more or less information... but we never withheld information from the patients.”

(N9)

#### 3.4.2. Healthcare Professionals

In emergency situations, the communication and actions were the same, i.e., they were not affected. Everyone indicated that they had to acquire new knowledge against the clock due to the urgent situation.

When speaking about the relationship with their colleagues, they agreed that during the pandemic, a greater camaraderie was observed. The group cohesion was generally strengthened:

“More bonding between colleagues, to a greater extent in the ICU because we were all new and without knowledge. We had great support from the referring nurses. We helped each other a lot in any way we could. It has been very intense and an exceptional experience with an incredible connection, very intense work shifts, and you saw your colleague who was the same as you. New knowledge at a rapid pace. Colleagues from another hospital came to help us.”

(N4)

“(…) it’s hard to forget, it was everything, 12-h shifts ‘to the death,’ but you had your colleague there, and with just one look, you knew that she was feeling the same way, but we supported each other, the camaraderie was spectacular and hard to forget, for me, these will remain in my heart for the rest of my life (…)”

(N5)

#### 3.4.3. Patients–Family

According to the interviewees, this was the most difficult part, as providing in-person care was very limited to avoid the propagation of the virus, resulting in a great deficit in communication. Differences were also found depending on the hospital service; for example, hospital floors and ICUs had resources, such as telephones or tablets for the patients to make video calls with their family members, but in the emergency department, this was more complicated although the family was always informed about the status of the patient:

“We try to get them to communicate every day, especially those with limitations, by providing them with a tablet. Those who took a sudden turn for the worse did not have time to talk to anyone. Visits were not allowed to avoid the spread of infections, but in special situations, they were allowed, but the family member had to wear a PPE (to say goodbye, for example).”

(N2)

In difficult situations, for example, when patients had to be intubated, they were allowed to speak to their families beforehand as long as it was not an emergency situation:

“(…) for the patients who were conscious, we made sure they had a telephone or table to speak to their families (…)”

(N8)

“(…) it was very hard, because it’s not the same thing to see them on the screen than in person, because you cannot touch, it’s been a big problem (…)”

(N5)

#### 3.4.4. Family–Health Professionals

Communication was affected because it could not be in-person although more importance was given to providing information to the families via the telephone:

“(…) communication was promoted, with a greater number of telephone calls to the family to provide information although I think it worsened, as they could not be there in person (…)”

(N9)

“(…) the family members were a lot more understanding; after observing our working conditions, they waited patiently because we were doing everything possible (…)”

(M1)

“The relatives have been much more understanding when they see how and in what conditions we were working. They made the task much easier; they waited patiently because they knew we are doing our best. People were afraid and understood the situation. They were informed at all times, but people waited patiently and didn’t resent our work so much.”

(N8)

## 4. Discussion

The main findings of the present study show that from the point of view of nurses, communication was slightly affected during the pandemic. This was notably observed in the non-verbal type of communication, with verbal communication maintained and sometimes strengthened. Nevertheless, an adaptive evolution of the health professionals, the patients, and their families was observed. This evolution was determined by a greater amount of information available about the disease, which marked the difference with the first periods (waves) of virus propagation. Aside from the improvement in the infrastructures, the improved organization, the acquisition of equipment such as PPEs, and, of course, the massive vaccination of the population also played a role. The negative emotional aspects identified were associated with fear and uncertainty. Positive emotional aspects emerged from behaviors, such as resilience and camaraderie, which created strong bonds in critical care and emergency teams.

For this, the nurses were able to adapt to the circumstances, greatly using different technologies to ensure a more effective communication through voice calls and video calls to share current information with family members, thus improving the emotional well-being when the in-person visits were restricted. As highlighted in other studies, efficient health communication is a key factor in the fight against the COVID-19 pandemic [41,42,43,44].

One of the aspects highlighted in other studies was the lack of training in communication skills [11] and its influence on how the health professionals managed the difficult periods, such as giving bad news, making difficult decisions, dealing with family members separated from their loved ones to prevent virus propagation, or the limited resources [45]. This aspect was present in the opinions from all the nurses interviewed in this study and was overall associated with negative emotions derived from the making of decisions in difficult environments. Already in the year 2020, Back et al. [11] highlighted the challenges faced by doctors in relation to communicating with patients in a pandemic context. Aspects such as facilitating virtual goodbyes between family members and dying patients with restricted access or how to explain decisions about why a particular patient will not receive a scarce resource were affected. However, in our study, we found that the adaptation of nurses to the new situation and communication difficulties was remarkable.

The study showed that the main negative feelings of the nurses were fear, stress, and physical and psychological wear although other feelings predominated, such as uncertainty, insecurity, chaos, frustration, impotence, and worry. A qualitative systematic review found that nurses working on the front lines during the COVID-19 pandemic experienced psychological, social, and emotional distress [46], and during this time, the health professional’s stress increased in Spain [47]. It has been demonstrated that adequate training on communication skills could have a direct impact on the mental health of workers and especially against burnout syndrome [17,20]. It has also been observed that the impact of the communication skills of emergency professionals is correlated with the satisfaction perceived by the patients and family members [48] and that communication skills improve the self-efficiency in professional practice perceived by the nursing professionals themselves [49,50]. Aspects that could have influenced participants’ stress, such as, for example, having children or elderly people at home, did not appear in the results of this study. However, we understand that they could have had some influence even if it was not made explicit by the nurses. These aspects would be interesting to investigate in future research studies.

Wearing personal protective equipment had an impact on communication in healthcare settings. These results are similar to those obtained in other studies conducted during and before the COVID-19 pandemic [51,52,53,54]. The nurses in our study stated that the care provided to individuals with a hearing disability was negatively affected by the use of masks. This problem was also revealed in a study [55] showing that there was a current research gap with regards to healthcare workers wearing masks, as the ability of healthcare staff to successfully communicate with patients and with colleagues is jeopardized, which may adversely affect the efficiency, effectiveness, equitability, and, most notably, safety of therapeutic interventions.

Another aspect to consider was the management and providing of information to people who had a hearing disability during the hardest periods of the pandemic. We must not forget that the gravity of the situation did not allow tending to the communication needs of these individuals as needed, with the resulting discomfort of the health professionals and especially the patients. Aspects associated with the difficulties of people with disabilities in the era of COVID-19 have been highlighted in numerous studies [56,57]. It is highly important to consider individuals with disabilities in terms of equality independently of the context.

With all of this information, we believe that it is highly important to grant communication the status it deserves within the planning of training courses of health professionals in situations such as the one we are currently experiencing. Recent studies have shown the importance of human factors in the training of inter-disciplinary teams within the context of the COVID-19 pandemic [58], especially in communication [59].

Another interesting aspect to discuss is that which refers to the technologies used by professionals to facilitate the communication of patients with their families or even to communicate the status of patients to relatives. Our respondents mostly reported the use of mobile phone devices and tablets as facilitators of communication. Other studies reported similar results [60,61], with granting special importance when it came to long-distance communication between people, which would facilitate the avoidance of face-to-face communication, playing a fundamental role in the prevention of infections and in the management of the pandemic.

The creation of resilient attitudes in health professionals and the overcoming of communication barriers is another important finding of the present work. The adaptation of nurses to the situation of uncertainty was notable. A series of strategies were put into place to improve communication with the patients and between the health professionals themselves, with new communication codes and channels adapted to the situation. The study participants reported the lack of a coordinated institutional response to mitigate the potential emotional effects of the pandemic on workers and the lack of communication protocols (beyond the ingenious and spontaneous resources of the staff to be able to communicate). The literature reports that some hospitals with previous experiences in caring for patients with high contagiousness [62] launched strategies to create a culture of resilience to face the pandemic. These strategies were based on three principles: leadership, structured communication, and, finally, the creation of a continuum of support for staff within the organization.

### 4.1. Key Practice/Policy Implications

If we have learned anything from this pandemic, it is that the human capacity to adapt is impressive. Health workers have been able to cope with physical and psychological overload to an unprecedented degree. We assume that the managers of health institutions have also been under great pressure to cope with this situation. However, as an implication for current and future clinical practice, we recommend a coordinated institutional response to mitigate the potential emotional effects on workers by designing appropriate communication and emotional expression protocols.

Since the onset of this pandemic, the professionals themselves have been improvising communication strategies and have adaptively developed an exemplary manner of providing care. However, we believe that some of the organizational and institutional weaknesses that have been directly or indirectly exposed in this work could be improved, which would have mitigated the suffering of health workers and patients.

### 4.2. Limitations

The main limitation of the study is its local nature. Therefore, we cannot confirm that the external validity of the study is adequate enough to be able to extrapolate the results to other clinical contexts. However, we believe that it could be useful for other researchers when comparing the impact of the COVID-19 pandemic on the communication dimension of health professionals in hospital emergency services and intensive care units.

Another limitation of this study is that it focused on communication at the level of professionals and patients/families. This research could perhaps have benefited from a perspective that connects to the managerial/organizational level, leaving aside half of the factors that influence the work of the participants and those who have already been investigated in numerous works [63,64].

Another threat to the validity of the study could come from the influence that the current situation could have had on the opinions of the participants, which could be amplified by the emotional and physical overload of an overwhelming situation, such as the current pandemic. However, being a phenomenological study, it makes sense that what was expressed by the participants is valued and analyzed in its context (a phenomenological method grants importance to the experiences and perspectives of the participants themselves). This method is oriented towards the approach to reality, starting from the internal frame of reference of the individual.

More studies are needed to explore the perspective of patients and family members to obtain a broader view of the problem. The external validity of the study could be increased with more interviews in broader contexts (other hospitals and other regions).

## 5. Conclusions

The analysis of the 12 qualitative interviews conducted with nurses who worked with COVID-19 patients in emergency areas and/or ICUs indicated that their communication experiences during the pandemic deteriorated, especially in the initial stages (early waves) and in the non-verbal modality. The impact of these communication difficulties were observed in aspects related to human interaction and could have contributed to the deterioration of the working conditions and patient care, especially with the elderly with perceptual difficulties and disabled individuals.

Communication limitations were identified, and these were due to the barriers derived from the use of the PPE and the initial fear of infection. The care provided to individuals with a hearing disability was negatively affected by the use of masks.

The nurses interviewed highlighted negative emotional aspects associated with the deficit in communication, such as the influence of work overload on communication and on their psychological state. Nevertheless, positive emotions were also identified, especially due to the creation of mutual support and group cohesion spaces of the work teams during the pandemic. Communication was adaptive in most of the cases, and the health professionals did not experience an evident deterioration except in the non-verbal communication modality.

As an implication for current and future clinical practice, we recommend a coordinated institutional response to mitigate the potential emotional effects on workers by designing appropriate communication and emotional expression protocols.

## Figures and Tables

**Table 1:** Main characteristics of the participants.

Number Code/Sex	Workplace	Age	Experience (Years)	Hospital
N1/Female	Emergencies	28	3	H1
N2/Female	COVID Hospital Unit	37	8	H2
N3/Female	Emergency Radiology Diagnosis	30	5	
N4/Male	Emergencies	33	4	
N5/Male	Emergencies	27	6	
N6/Female	ICU	41	9	H3
N7/Female	ICU	31	4	
N8/Female	ICU	53	18	
N9/Female	ICU	32	6	
N10/Male	ICU	34	8	H4
N11/Male	ICU	33	5	
N12/Male	Emergencies	64	20	H5

**Table 2:** Script of questions and topics used in the semi-structured interviews.

Personal experience before the pandemic and initial experiences at the start of the pandemic
How would you describe your process of adaptation during the last few months until today? Please explain to me what the process entailed for you to go to work every day in an environment with COVID-positive patients
Feelings perceived during the different waves of the pandemic
How was the communication between the health professionals and the patients? What happened with the patients subjected to NIV *?
Please tell us about any experience about patients who had to be intubated quickly, without being able to say good-bye, if this occurred in your presence
Do you think the patient received the same amount of information about his/her disease as if the pandemic had not occurred?
Can you describe the communication between health professionals during the pandemic? Do you think communication has been negatively affected?
Before the pandemic, do you think you could have better interpreted the mood of a colleague at the hospital?
Do you think the relationships between colleagues have been affected by the pandemic? Explain your answer
What advice would you give to a colleague who is starting work at the same service you are at in order to effectively communicate with patients and the other health professionals?
After the workday, when you are at home, have you had to deal with some feelings? How would describe it/them?
As for the training for dealing with the pandemic, what aspects would you highlight?
Have you dealt with patients with a communication difficulty or with people who have some type of limitation or disability? Do you think the distancing protocols and personal protection equipment have had an influence on communication?
Have you noticed some alterations in communication with respect to the patient’s families and the health professionals during the pandemic?

* NIV, non-invasive ventilation.

**Table 3:** Main findings of the study.

Dimension	Category	Sub-Category
Communication	Communicative expression	Verbal: - Verbal communication was affected, especially at the beginning of the pandemic, due to the use of personal protection equipment (mask, screen, robe, etc.) although it is precisely due to this that communication improved over time and was re-enforced between colleagues by the surge in empathetic feelings. - Communication by telephone to contact the family was used more than before. - The nurses believed that the patients received the same amount of information about their disease. - The health professionals who worked directly with COVID-positive patients, at which time a colleague was present to “mirror” them, had better work organization. - Communication in situations of emergency was not affected, independent of whether the patient was COVID-19 positive.
		Non-verbal: - Non-verbal communication was more complicated due to the use of PPE (mask). - Facial expression was limited so that their attention was more focused on the expression of the eyes or gestures. - The participants agreed that before the pandemic, they could better observe the mood of a colleague as compared to the present time due to the use of masks. However, at present, they could still detect the emotions of others as long as they focused their attention on the eyes and gestures.
	Limitations	- Various limitations were found, among which we found elderly who were deaf, people with language barriers, patients who were under non-invasive ventilation, patients in the process of weaning, patients who had undergone a tracheostomy, etc. - Other means of communication were utilized for better communication, such as gestures or using paper and pen to write down what they wanted to say.
Emotional aspects	Positive	- The participants underlined positive feelings, such as camaraderie or the feeling of becoming close to their colleagues, and other feelings were found, such as the acquisition of new knowledge and the feeling of overcoming, the special feeling established with the “mirror” colleague, and feelings of solidarity and responsibility.
	Negative	- The main negative feelings were fear, stress, and physical and psychological wear although other feelings predominated, such as uncertainty, insecurity, chaos, frustration, impotence, and worry.
Overload	First wave	- Lack of PPE, lack of information, greater exposure, greater psychological difficulty, chaos, and in Primary Care, only the more urgent pathologies were tended to in person.
	Second wave	- The participants had more information, more equipment, and experience. There was less fear and more protocols although these were still changing. The nurses became accustomed to wearing masks and PPE.
	Third wave	- The participants characterized it as the hardest period of work due to the pressure on medical care although they were more accustomed to wearing PPE and worked faster. - The nurses had more knowledge, and things were more peaceful thanks to the start of vaccination. They agreed that they felt safer due to the use of the PPE.
Relationships	Professionals–patients	- Loss of physical contact and a much more limited communication due to the use of PPE, especially utilized to reduce the virus exposure time. - With time, the relationships evolved given the increased knowledge and therefore increased safety. - The general perception was that the patients received the same amount of information about their disease, without considering the emotional part (which was impaired). - Communication with patients under NIV or with some type of disability was more difficult.
	Healthcare professionals	- In emergency situations, the communication and actions were the same. - Nurses indicated that they had to acquire new knowledge against the clock due to the situation. - When speaking about the relationship with their colleagues, they agreed that during the pandemic, a greater camaraderie was observed. The group cohesion was generally strengthened.
	Patients–family	- This was the most difficult part, as providing in-person care was very limited to avoid the propagation of the virus, so that a great deficit in communication was observed. - We also found differences depending on the hospital service (e.g., hospital floors and ICUs had resources, such as telephones or tablets for the patients to make video calls with their family members, but in the emergency department, this was more complicated). - In difficult situations, for example, when patients had to be intubated, they were allowed to speak to their families beforehand as long as it was not an emergency situation.
	Family–professionals	- Communication was affected because it could not be in-person although more importance was given to providing information to the families via the telephone.

## Data Availability

The data are available upon email request to the corresponding authors.
